# The State and the Malingerer

**Published:** 1918-08-24

**Authors:** 


### THE SIMULATION OF DISEASE.

### I. The State and the Malingerer.

IN every community so far developed as to impose obligations and restraints upon its members there must arise occasions when the demands of the State are opposed to the inclinations of the individual. In such a conflict the struggle is one of force against cunning, and in this manner we may suppose the earliest attempts at feigning disease to have arisen. That it was common in Greece is shown by the repeal of the death penalty by Charondas for those who sought by this means to evade military service, who contented himself with exposing them upon a scaffold for three days clad in female raiment.

In more modern times the motives increase in complexity. Monetary considerations, the evasion of punishment or unpleasant tasks, the desire for sympathy of the *malade imaginaire*, the imitative impulse of the unstable-minded, all furnish numerous examples. Even the infirmities of the great have been copied by sycophants desirous of flattering an exalted personage.

Thus Louis XIV.—*le grand monarque*—was surrounded by a host of courtiers who feigned to be afflicted with a similar malady—*fistula in ano*. Many of these gentry, however, met with a fitting punishment, for, falling into the hands of ignorant practitioners, who pierced the intestine, they really acquired the disease.

The Workmen's Compensation Act, and later the National Insurance Acts, naturally opened up a wide field for the shirker and impostor. But whether pure malingering—and by this we mean malingering without any substratum of real disease—is commoner than it was, is far from proved.

### IS MALINGERING ON THE UP-GRADE?

Many observers have noted the increase of recent years in certain nerve disorders—traumatic neurasthenia, hysteria, and the like—and some attribute it directly to the pernicious influence of the Compensation Act. But against such a view several considerations may be urged. The increasing strain of modern industrial conditions speedily finds the weak spot in a man's constitution. Thus Oppenheim states that a far higher percentage of syphilitics develop *tabes* than of yore. Alcoholism, too, is certainly more prevalent among certain classes, *e.g.* women industrial workers. Again, the great strides that have been made in the diagnosis and classification of nerve disorders has led to the recognition of many functional conditions that would previously have been placed in the comprehensive pigeon-hole of Hysteria. Twenty-five years ago Charcot observed that the idea of malingering was only too often based on the ignorance of the doctor, and though probably less true to-day, advancing knowledge will doubtless reveal cases of medical injustice. Moreover, the Insurance Act of 1911 embraced all grades of the industrial community, many of whom (notably the women) had never previously enjoyed any sickness benefit. Indeed, the ill-health of the female worker reached a figure far exceeding all actuarial estimates. The clubs and Friendly Societies touched only the more prosperous fringe of the artisan population, and many members took a pride in never availing themselves of benefits. The accession, therefore, of millions of insured persons for whom no relative standard of health existed, naturally upset established criteria. Nevertheless, the Insurance Commissioners state that although " we find little evidence of fraud on the part of insured persons, or of deliberate malingering, there is considerable evidence of (*a*) a tendency to take the utmost advantages of the benefits under the Act, (*b*) a tendency to complain of trivial complaints, (*c*) a tendency to prolong unduly the period for which members remain on the fund, especially during periods of unemployment."

*Malingering.* By A. Bassett Jones and Llewellyn J. Llewellyn. (London: William Heinemann. 25s. net.)

### A PREMIUM ON DISHONESTY.

A brief consideration of the Workmen's Compensation Act and the Insurance Acts will show us why this should be so. Under the former an injured workman gets nothing for the first week of incapacity, provided he returns to work on or before the seventh day; up till the fourteenth day he can only claim for the days of incapacity over seven; *i.e.*, if he returns on the tenth day after injury he can claim three days' compensation. If, on the other hand, his incapacity lasts for more than fourteen days, he is paid compensation for the whole period of disablement. A more deliberate incentive to prolong the disablement period can hardly be conceived, and there is no doubt that it has had this effect. Thus during the period 1908-1913 the number of non-fatal accidents increased six times as rapidly as the number of fatal accidents, and of this increase the bulk consisted of injuries lasting *from two to three weeks*. In 1908, the year of the introduction of the Act, the number of accidents was 78,000; in 1913, 136,000; although the number of workpeople employed was almost exactly the same in both years.

Again, under National Insurance no sickness benefit can be obtained before four days have elapsed, thus directly encouraging a habit of valetudinarianism, if nothing worse. Over-insurance, too, is another of the temptations to prolong indefinitely some minor ailment. By belonging to several clubs, or by payments to the voluntary contributor's section of his approved society, a man can often be in receipt of a larger income when on the sick list than when in full work. The *impersonality* of a Government department, the feeling that so much has been paid in, therefore something should be got out, the temptation to go sick when out of work, are doubtless all contributory factors. This state of affairs has been strikingly illustrated in the case of the French

**The Simulation of Disease**—(*continued*).

railways. Prior to their nationalisation in 1909, there were 474,000 days of sickness; in 1911, after the change, the figure had risen to 656,000; in other words, no fewer than 54 per cent. of the employees were ill at one time or another.

THE DOCTOR'S DILEMMAS.

The position of the medical man with regard to his insured patients is not an easy one. In the old club days an honest opinion as to the real cause of incapacity from the medical man, or the refusal to grant a certificate, was often sufficient to lose him the entire club.

State Insurance would, it was hoped, by rendering the doctor independent of his patients' whims and caprices, enable him to treat them without fear or favour. But the rush of panel work is so great and the remuneration so small that the medical man is often enough obliged to take the patients' statements without question. A doctor in a district gets a reputation for complaisance in the matter of certificates; the patients flock to him, the ease with which an insured person can transfer himself from one doctor to another rendering this possible. We do not wish to suggest that the granting of misleading certificates is a widespread practice. That it does occur anyone with experience of Insurance work would be the first to acknowledge. But that the medical man acts from dishonest motives save in a negligible minority of cases we cannot believe. The fault lies deeper than a mere desire to be rid of a troublesome patient. It is to be found rather in social conditions. The medical man is debarred from prescribing what the patient really needs—good food, decent housing and sanitation, lighter work, freedom from worry.

Having thus considered, in brief, some of the factors which, in civil life, tend to produce an increase, if not of true malingering, at least of an unnecessary prolongation of industrial incapacity, let us proceed to analyse some of the main features that should guide us to a just conclusion.

A CLASSIFICATION OF MALINGERERS.

The type of person with whom we are dealing must first be considered.

He may, of course, be of normal mentality. These patients, curiously enough, are as a rule easy of detection. They lack tenacity of purpose, the motive is often obvious, the deceit relinquished as soon as the desired end has been gained.

Of a far different stamp is the degenerate malingerer, the type found in prisons, reformatories, and workhouses. Preferring idleness to a decent living, the deceits he practises are often elaborate, and no pains are too great if he can convince the physician of the truth of his assertions. Thus one feigned epileptic suffered knives to be thrust beneath his nails and irritating powders blown into his eyes without flinching. He even fell a distance of thirty feet to convince the expert, though he subsequently admitted his deceit.

A third class is the hysterical patient whose symptoms vary from day to day; often puzzling to the physician at first from their failure to conform with those of any known disease. But a little experience and a keen eye for type rarely mislead one to confound hysterical manifestations with genuine organic disease. Their ready suggestibility, and the exaggerated gestures with which they describe some trifling ailment are perfectly characteristic. To what extent such cases can be dubbed malingering is a question into which we can enter but briefly here, and the reader would do well to peruse Babinski's fascinating monograph on the subject.

Yet a fourth group is formed by the weak-minded or insane malingerer. Insanity may, of course, be feigned by the person of perfectly sound mind (as, for instance, David before Achish, King of Gath); but the bulk of cases of feigned insanity occur among epileptics, paranoiacs, and weak-minded criminals, who assume this plea to evade punishment. In the investigation of such cases even the alienist must tread delicately, for of these borderline states of mental instability we have yet much to learn. (*To be continued.*)

